# Multidimensional patterns of malocclusion in treatment-seeking Turkish orthodontic patients: A retrospective cross-sectional study

**DOI:** 10.1097/MD.0000000000051302

**Published:** 2026-10-23

**Authors:** Merve Berika Kadıoğlu, Nazlı Türkel, Meliha Rübendiz

**Affiliations:** a Department of Orthodontics, Faculty of Dentistry, Ankara University, Ankara, Türkiye; b Department of Orthodontics, Graduate School of Health Sciences, Ankara University, Ankara, Türkiye.

**Keywords:** artificial intelligence, crossbite, epidemiology, malocclusion, orthodontics, overbite, prevalence

## Abstract

Malocclusion is a common craniofacial disorder with significant functional and aesthetic implications. Although sagittal, vertical, and transverse discrepancies frequently coexist, most prevalence studies evaluate these traits separately. This study aimed to analyze the prevalence and multidimensional interactions of sagittal, vertical, and transverse malocclusions in a large Turkish treatment-seeking orthodontic cohort using artificial intelligence-assisted overbite measurements for standardized vertical assessment. A retrospective cross-sectional analysis was performed on standardized intraoral photographs and cephalometric radiographs of 2624 treatment-seeking orthodontic patients (1125 males and 1499 females) treated at a tertiary referral university clinic between 2007 and 2020. Sagittal malocclusions were classified according to Angle’s classification (class I, II, and III). Transverse relationships were defined by the presence or absence of posterior crossbite, while vertical subgroups were established using artificial intelligence-assisted overbite measurements for standardized vertical assessment. Chi-square tests with Bonferroni-adjusted pairwise comparisons and multinomial logistic regression were performed. Class II malocclusion was the most prevalent sagittal malocclusion (49.1%), followed by class I (33.0%) and class III (17.9%). Normal transverse relationships predominated in class I (54.3%) and class II (66.9%), whereas more than half of class III individuals exhibited posterior crossbite (57.9%). In class I and II, posterior crossbite was seen more frequently in open bite cases and was least common in deep bite cases. In class III, posterior crossbite prevalence remained high across all vertical subgroups, regardless of overbite pattern. Multinomial logistic regression showed that deep bite had the strongest independent association with class II malocclusion, whereas bilateral posterior crossbite had the strongest independent association with class III malocclusion. The prevalence of orthodontic malocclusions should be evaluated using a multidimensional approach that considers the coexistence of sagittal, vertical, and transverse discrepancies rather than assessing each occlusal dimension independently. The strong association between class III malocclusion and posterior crossbite, together with the vertical–transverse interactions observed in class I and II, highlights the importance of comprehensive multidimensional orthodontic assessment and individualized treatment planning. Recognizing these multidimensional patterns may facilitate comprehensive clinical assessment and guide future epidemiological research.

## 1. Introduction

Malocclusion, defined as a misalignment between the upper and lower dental arches, is one of the most common developmental disorders of the craniofacial complex, affecting mastication, speech, esthetics, and quality of life.^[1,2]^ Although Angle’s classification, proposed in the late 19th century, remains the most widely applied system in both clinical and epidemiological research,^[3]^ it focuses exclusively on sagittal molar relationships and fails to capture the multidimensional nature of malocclusion.^[4,5]^ Contemporary orthodontics recognizes that sagittal, vertical, and transverse deviations rarely occur in isolation but instead interact dynamically, influencing diagnosis, treatment planning, and prognosis.^[6]^ With the advancement of cephalometric and digital imaging, it has become clear that a purely sagittal perspective is insufficient to describe occlusal complexity, highlighting the need for a multidimensional approach to ensure diagnostic accuracy and long-term stability.^[4]^

In ideal occlusion, the maxillary arch surrounds the mandibular arch with harmonious sagittal alignment, normal overbite and overjet, and balanced transverse widths.^[5]^ This relationship is essential for efficient mastication and normal oral function.^[7]^ However, sagittal malocclusions – Angle class I, II, and III – frequently coexist with vertical deviations ranging from open bite to deep bite, as well as transverse discrepancies in the posterior segments, including unilateral or bilateral crossbites.^[8]^ Clinical and morphometric studies confirm these interrelationships: posterior crossbite, indicating maxillary constriction, is common in class III malocclusion^[9]^; open bite is often associated with maxillary constriction^[10]^; while deep bite has been linked to strong masticatory musculature and relatively preserved transverse development.^[11]^ Such interrelationships highlight the importance of comprehensive multidimensional assessment in both epidemiological research and clinical orthodontic practice.^[12]^

The etiology of malocclusion is multifactorial, involving genetic predisposition, functional, and environmental influences such as abnormal oral habits, respiratory dysfunction, trauma, and premature tooth loss.^[6,13,14]^ These factors affect the dento-craniofacial complex not at a single point, but across sagittal, vertical, and transverse dimensions. Through functional adaptations, teeth, skeletal structures, and soft tissues acquire new morphologies, leading to diverse craniofacial presentations. Consequently, orthodontic malocclusions, which are recognized by the World Health Organization as one of the most prevalent oral health conditions worldwide, should be regarded as a multidimensional public health concern rather than a single-plane disorder.^[1]^

With rising aesthetic awareness and increased demand for orthodontic care, understanding the frequency and complexity of malocclusions has become even more crucial to evaluate disease burden.^[15,16]^ This requires prevalence studies that go beyond sagittal evaluation alone and also address vertical and transverse traits in an integrated manner.

Epidemiological studies play a critical role in identifying the extent and severity of such public health problems, prioritizing healthcare needs and guiding future research and service planning. Understanding the prevalence of malocclusions is vital for both clinicians and public health authorities to assess current challenges, determine associated risk factors, and develop appropriate strategies for assessment and management.^[17]^ However, despite the multidimensional nature of malocclusion, most epidemiological studies have evaluated each occlusal plane independently, and the coexistence of sagittal, vertical, and transverse discrepancies has rarely been investigated.

Most existing prevalence studies in different populations focus on a single occlusal dimension – most often the sagittal – and neglect how deviations across planes may compound 1 another or interact.^[1,17–25]^ Furthermore, vertical discrepancies are typically assessed via subjective visual inspection, and few studies explore the co-distribution of malocclusion traits across planes. Moreover, transverse parameters are often simplified as the presence or absence of posterior crossbite, without accounting for the influence of sagittal and vertical morphology.^[23,24]^ This approach limits both the accuracy of prevalence estimates and the ability to draw clinically relevant conclusions. Consequently, the overall severity and complexity of malocclusion patterns often remain underrepresented.

Advances in artificial intelligence (AI) have introduced new opportunities for more standardized and objective data analysis in large samples. AI-assisted cephalometric tools provide automated and reproducible landmark identification and linear measurements, thereby reducing examiner-dependent variability, especially in vertical assessment.^[26]^ These AI-assisted tools are designed to support, rather than replace, clinician-guided measurements and provide a practical alternative for retrospective analyses where manual measurements are time-consuming and 3-dimensional imaging such as cone beam computed tomography is impractical because of ethical and financial considerations.^[27]^ Nevertheless, few studies have integrated AI-assisted methods into multidimensional epidemiological analyses of malocclusion.

Given these gaps, the aim of this retrospective cross-sectional study was to evaluate multidimensional malocclusion patterns in a large cohort of treatment-seeking Turkish orthodontic patients. Using standardized photographic assessment together with AI-assisted overbite measurements, the study aimed to characterize the prevalence and interrelationships of sagittal, transverse, and vertical malocclusion traits.

Unlike previous studies that evaluated each occlusal plane separately, this multidimensional approach facilitates the identification of clinically relevant coexistence patterns and better reflects the complexity of malocclusions encountered in routine orthodontic practice. The findings are expected to provide clinically relevant epidemiological evidence that may support diagnosis, treatment planning, and future research on multidimensional malocclusion patterns.

## 2. Materials and methods

### 2.1. Study design, ethical approval, and statements

This retrospective cross-sectional prevalence study evaluated the distribution of orthodontic malocclusions in treatment-seeking orthodontic patients attending a tertiary referral university clinic. Pretreatment standardized intraoral photographs and cephalometric radiographs of patients seeking orthodontic treatment at the Department of Orthodontics, Faculty of Dentistry, Ankara University, a tertiary referral center in Türkiye between 2007 and 2020 were analyzed. Before starting the study, ethical approval was obtained from the Ethics Committee of Ankara University Faculty of Dentistry (approval no. 36290600/80/2024; May 20, 2024). The study protocol adhered to the ethical principles of the World Medical Association Declaration of Helsinki for research involving human participants.

Written informed consent for the academic use of anonymized clinical records was obtained from all participants before treatment as part of standard clinical procedures. For participants younger than 16 years, consent was provided by parents or legal guardians. The study was conducted in accordance with the Strengthening the Reporting of Observational Studies in Epidemiology guidelines for cross-sectional studies.

### 2.2. Eligibility criteria

Patient records were screened consecutively according to the predefined eligibility criteria. Patients who underwent orthodontic treatment and met the following inclusion criteria were included in the present study:

- No craniofacial deformities or syndromes
- Age ≥7 years
- Fully erupted 1st permanent molars and incisors
- No multiple missing teeth
- No history of orthodontic treatment
- High-quality lateral cephalometric radiographs and intraoral photographs

Patients meeting the following exclusion criteria were not included:

- Multiple tooth deficiencies, such as hypodontia or oligodontia
- Presence of craniofacial syndromes or deformities
- Previous orthodontic treatment
- Incomplete, nonstandardized, or low-quality photographic or radiographic records

### 2.3. Sample size calculation

A total of 3720 consecutive patient records were initially screened between 2007 and 2020. As the study was designed to evaluate the entire eligible population rather than a selected sample, no a priori sample size calculation was necessary. Owing to the inclusion of all eligible cases that met the inclusion criteria within the specified time period, no sampling strategy was applied. Therefore, the study was based on a census of all eligible treatment-seeking orthodontic patients within the study period rather than on a sampled population, making an a priori sample size calculation unnecessary.

Records were sequentially assessed according to the predefined eligibility criteria. Patients with previous orthodontic treatment, craniofacial syndromes or deformities, multiple missing teeth, age younger than 7 years, incomplete eruption of the 1st permanent molars or incisors, or incomplete/nonstandardized photographic or radiographic records were excluded. After eligibility assessment, 2624 patients (1125 males and 1499 females) who met the inclusion criteria were included in the final analysis. The participant selection process is summarized in Figure 1, including the numbers of screened, excluded, and included records together with the predefined reasons for exclusion. The final treatment-seeking orthodontic cohort consisted of patients aged 7 to 25 years (mean age: 14 ± 4.23 years), representing the typical age range of orthodontic populations that include both growing and young adult patients.

**Figure 1.**
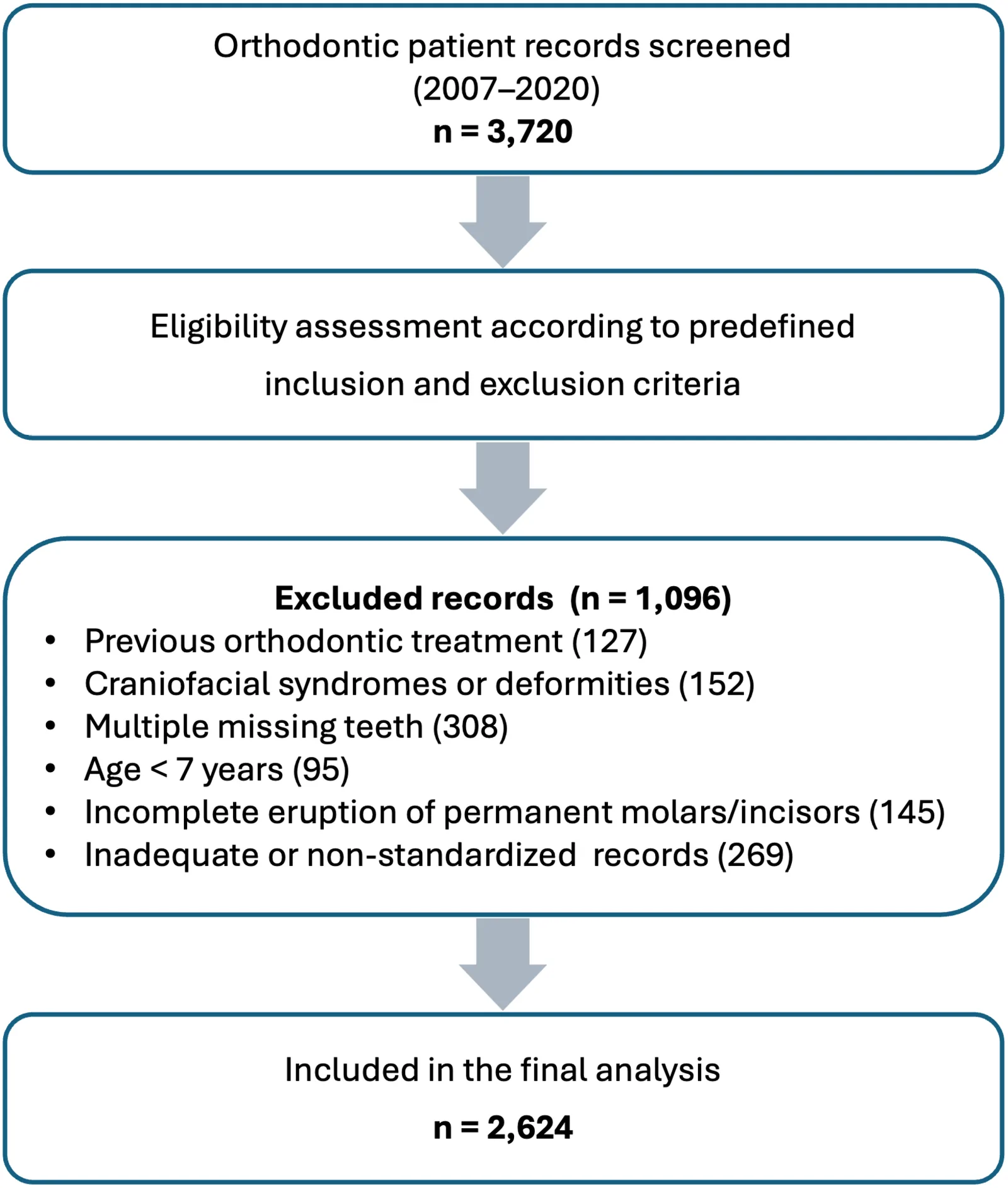
Flow diagram of participant selection. A total of 3720 consecutive patient records screened between 2007 and 2020 were assessed for eligibility according to the predefined inclusion and exclusion criteria. Overall, 1096 records were excluded for the reasons shown, and 2624 patients were included in the final analysis.

No missing data were present in the final analytical dataset, as records with incomplete or nonstandardized photographic or radiographic documentation had been excluded during the eligibility assessment.

### 2.4. Data collection and formation of study groups

Standardized intraoral photographs were used for the classification of malocclusions in the sagittal and transverse directions. This approach is supported by previous validation studies demonstrating high reliability for molar classification and occlusal trait detection.^[28]^ All photographs were obtained by a single calibrated dental technician using a Nikon D90 DSLR camera equipped with a 100-mm macro lens under standardized lighting conditions, camera-to-subject distance, and angulation. This protocol ensured high reproducibility and minimized variability in transverse evaluation across the entire dataset. Classification was performed visually by an experienced orthodontist following photographic calibration procedures.

Sagittal malocclusion was 1st classified using standardized intraoral photographs. Patients were subsequently classified as class I, class II, or class III according to the sagittal relationship of the 1st permanent molars using Angle’s classification.^[3]^ In addition, gender distribution within each sagittal group was determined separately.

Transverse occlusal relationships were evaluated hierarchically. First, patients were categorized according to the presence or absence of posterior crossbite. Cases with posterior crossbite were subsequently subclassified according to the extent of the transverse discrepancy into 3 categories: single-tooth, unilateral, and bilateral posterior crossbite. Accordingly, 4 transverse occlusal subgroups were analyzed within each sagittal malocclusion group as follows:

- Non-crossbite (normal occlusal relationship): The buccal cusps of the upper posterior teeth are located on the buccal side of the antagonist lower teeth.
- Single-tooth posterior crossbite: Only 1 upper posterior tooth (right or left) is positioned lingually to its antagonist lower tooth.
- Unilateral posterior crossbite: Two or more upper posterior teeth on 1 side are positioned lingually to the antagonist lower teeth.
- Bilateral posterior crossbite: Two or more upper posterior teeth on both sides are positioned lingually to the opposing lower teeth.

Vertical occlusal relationships were subsequently assessed from cephalometric radiographs rather than photographs by measuring dental overbite, which represents the degree of vertical overlap of the incisal edges. Overbite measurements were performed using WebCeph (AssembleCircle Corp.), a web-based AI-assisted cephalometric analysis platform. The software automatically identified cephalometric landmarks and calculated the vertical relationship as millimetric overbite. To ensure measurement accuracy, every automated landmark was visually verified by a calibrated orthodontist. In cases of incorrect AI landmark detection, manual correction was applied before final measurement. The midpoint between the central incisors was used as a reference in cases of severe crowding or rotation. Overbite measurements were primarily based on the incisal edges of the maxillary and mandibular central incisors. In cases of mild crowding or rotation, the midpoint between the central incisors was used as the reference. When severe displacement or rotation of a central incisor could compromise reliable midpoint determination, the markedly displaced tooth was not used as the reference for overbite measurement. Instead, the reference point was established according to the overall anatomical alignment of the anterior dentition following manual landmark verification and correction. No patients were excluded on this basis.

According to orthodontic standards, the normal overbite value is approximately 2 mm, with an acceptable variation of ±2 mm.^[2,29]^ Accordingly, individuals in each transverse subgroup were categorized into 3 vertical subgroups based on overbite measurements as follows:

- Open bite: Overbite <0 mm
- Normal overbite: Overbite = 0–4 mm
- Deep bite: Overbite >4 mm

To evaluate the reliability and validation of the measurements, an additional analysis was performed using a representative subsample of 50 randomly selected patients. For validation of the AI-assisted overbite measurements, overbite was independently measured using both the AI-assisted WebCeph platform (AssembleCircle Corp.) and conventional manual cephalometric tracing performed in Dolphin Imaging software (version 11.95; Dolphin Imaging & Management Solutions) by an experienced orthodontist. The conventional manual measurements were performed independently, with the cases assessed in a randomized order and the observer blinded to the AI-assisted measurement results. Agreement between the 2 methods was assessed using an absolute-agreement, single-measure intraclass correlation coefficient. In addition, inter-observer agreement for sagittal and transverse malocclusion classifications was evaluated by independently reassessing the same subsample by a 2nd experienced orthodontist. Agreement between the initial and repeated classifications was assessed using Cohen’s kappa coefficient.

Consequently, the analyses were performed sequentially. First, sagittal malocclusion prevalence was determined. Second, sex distribution within each sagittal group was evaluated. Third, transverse occlusal relationships were analyzed within each sagittal malocclusion group. Finally, transverse and vertical occlusal relationships were evaluated simultaneously within each sagittal malocclusion group.

### 2.5. Statistical Analysis

Statistical analyses were performed using IBM SPSS Statistics for Windows, Version 26.0 (IBM Corp.). Categorical variables were presented as frequencies and percentages. Pearson’s chi-square test was used to evaluate the distribution of sagittal malocclusion according to sex, differences in transverse occlusal relationships among sagittal malocclusion groups, and differences in transverse occlusal relationships among vertical occlusal subgroups within each sagittal malocclusion group. When the overall chi-square test was statistically significant, Bonferroni-adjusted pairwise comparisons were performed to identify the source of the differences. To complement significance testing, Cramer’s *V* was calculated to quantify the strength of statistically significant associations and interpreted as indicating small, moderate, or large effect sizes.

To further examine the independent associations of age, transverse occlusal relationship, and vertical occlusal relationship with sagittal malocclusion, multinomial logistic regression analysis was performed. Sagittal malocclusion (class I, class II, and class III) was entered as the dependent variable, with class I serving as the reference category. Age was included as a continuous variable, whereas transverse and vertical occlusal relationships were entered as categorical independent variables, using non-crossbite and normal overbite as the respective reference categories. Regression results were expressed as odds ratios (ORs) with 95% confidence intervals (95% CIs). Model fit was evaluated using the likelihood ratio chi-square test together with the Cox and Snell and Nagelkerke *R*^2^ statistics. A 2-sided *P* value <.05 was considered statistically significant.

## 3. Results

This study evaluated the standardized intraoral photographs and lateral cephalometric radiographs of 2624 treatment-seeking orthodontic patients (1125 males and 1499 females) who attended the Department of Orthodontics between 2007 and 2020 and met the predefined eligibility criteria.

The frequency distribution of sagittal malocclusions according to Angle’s classification is presented in Table 1 and Figure 2. The prevalence of class I, class II, and class III malocclusions was 33.0% (n = 866), 49.1% (n = 1288), and 17.9% (n = 470), respectively. The distribution of sagittal malocclusions did not differ significantly between females and males (χ^2^ = 3.598, *P* = .168).

**Table 1:** Distribution of sagittal malocclusion according to sex and results of the Pearson chi-square analysis.

Gender	Sagittal malocclusion						Total	χ^2^	*P*
	Class I		Class II		Class III				
	n	(%)	n	(%)	n	(%)	n		
Female	495	33.1	753	50.2	251	16.7	1499	3.598	.168
Male	371	33.0	535	47.6	219	19.4	1125		
Total	866	33.0	1288	49.1	470	17.9	2624		

*P* values were calculated using the Pearson chi-square test. Statistical significance was set at *P* < .05.

% = percentage, n = number of participants, χ^2^ = Pearson chi-square statistic.

**Figure 2.**
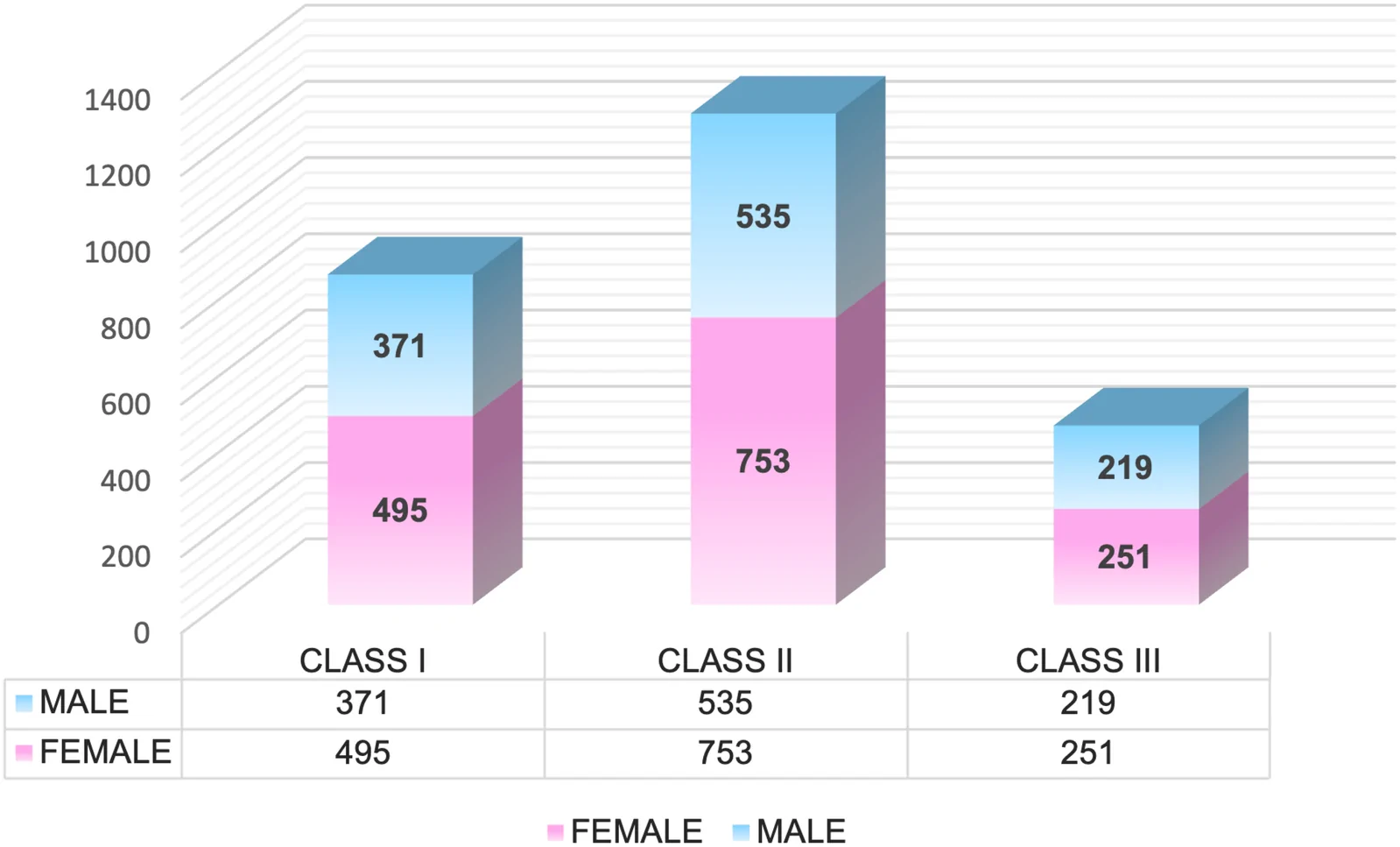
Distribution of sagittal malocclusion by sex.

The distribution of sagittal malocclusions in relation to transverse occlusal relationships is shown in Table 2 and Figure 3. Percentages in the vertical columns represent the distribution of each transverse occlusal relationship (non-crossbite, single-tooth posterior crossbite, unilateral posterior crossbite, or bilateral posterior crossbite) within each sagittal group (class I, II, or III). Differences in these distributions among sagittal groups were assessed using the chi-square test, with Bonferroni correction applied for pairwise comparisons (*P* < .05). A significant association was observed between sagittal malocclusion and transverse occlusal relationship (χ^2^ = 562.45, *P* < .001), with a moderate effect size (Cramer’s *V* = 0.327). Bonferroni-adjusted pairwise comparisons demonstrated that non-crossbite and bilateral posterior crossbite differed significantly across all 3 sagittal malocclusion groups. Additionally, single-tooth posterior crossbite was more frequent in class I, whereas unilateral posterior crossbite was more frequent in class III. Significant pairwise differences identified by the Bonferroni correction are indicated by different lowercase superscript letters in Table 2.

**Table 2:** Distribution of transverse occlusal relationships among sagittal malocclusion groups and results of the Pearson chi-square analysis.

Transverse occlusal relationship	Class I n (%)	Class II n (%)	Class III n (%)	Total n (%)	χ^2^	*P*	Cramer’s *V*
Non-crossbite	470 (54.3)^a^	862 (66.9)^b^	121 (25.7)^C^	1453 (55.4)	562.45	<.001	0.327
Single-tooth posterior crossbite	252 (29.1)^a^	244 (18.9)^b^	77 (16.4)^b^	573 (21.8)			
Unilateral posterior crossbite	80 (9.2)^a^	134 (10.4)^a^	89 (18.9)^b^	303 (11.6)			
Bilateral posterior crossbite	64 (7.4)^a^	48 (3.7)^b^	183 (38.9)^C^	295 (11.2)			
Total	866 (100)	1288 (100)	470 (100)	2624 (100)			

Percentages represent the distribution of transverse occlusal relationships within each sagittal malocclusion group. Different lowercase superscript letters (a, b, and c) indicate significant pairwise differences according to the Bonferroni correction (*P* < .05).

% = percentage, Cramer’s *V* = effect size, n = number of participants, χ^2^ = Pearson chi-square statistic.

**Figure 3.**
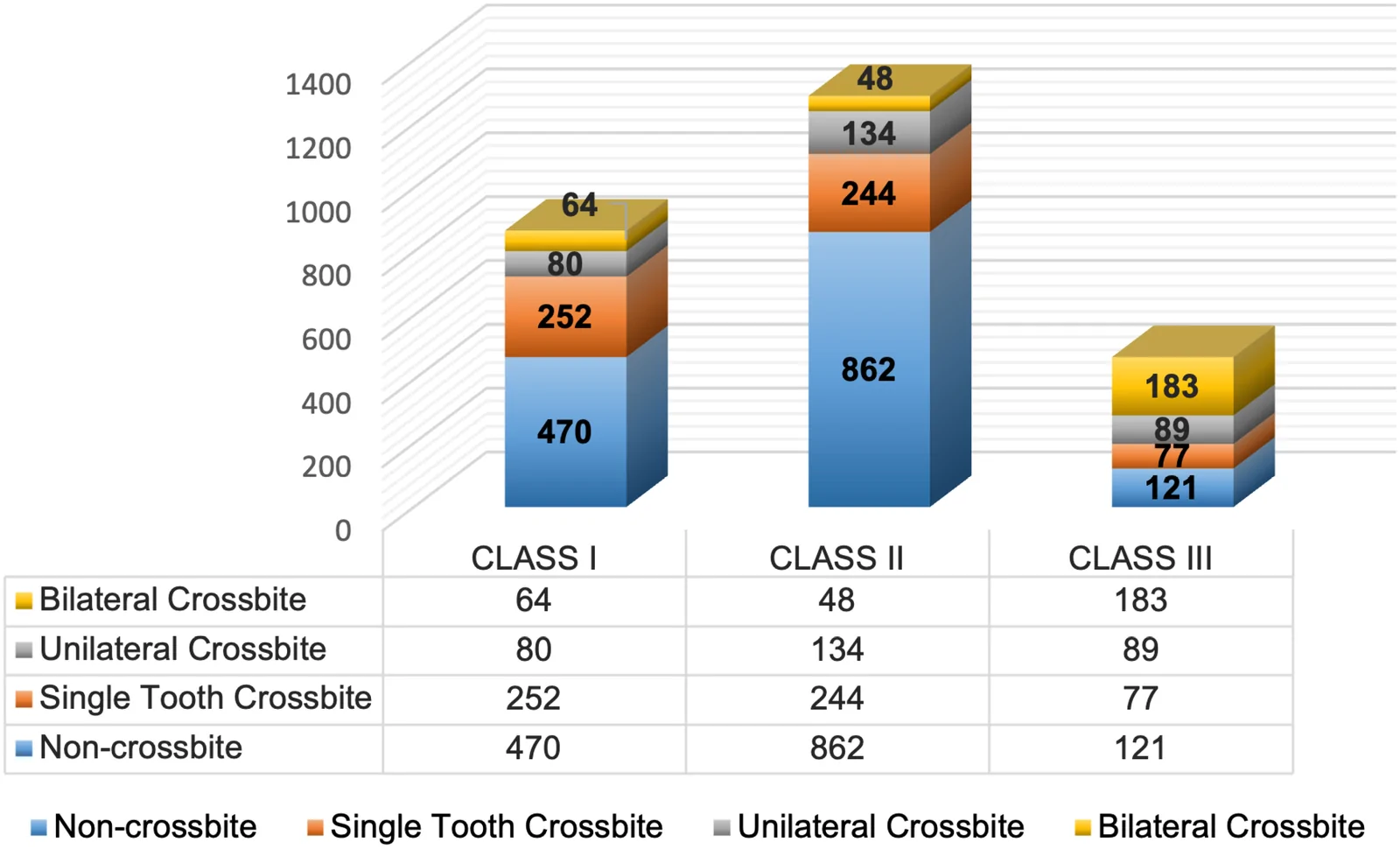
Distribution of transverse occlusal relationships (non-crossbite, single-tooth posterior crossbite, unilateral posterior crossbite, and bilateral posterior crossbite) among sagittal malocclusion groups (class I, II, and III).

Findings of the combined transverse and vertical occlusal relationship analyses are presented separately for each sagittal malocclusion group: class I (Table 3), class II (Table 4), and class III (Table 5). In Tables 3–5, percentages represent the distribution of transverse occlusal relationships (non-crossbite, single-tooth posterior crossbite, unilateral posterior crossbite, and bilateral posterior crossbite) within each vertical occlusal subgroup (open bite, normal overbite, and deep bite). Differences among vertical subgroups were evaluated using the Pearson chi-square test. When statistically significant, Bonferroni-adjusted pairwise comparisons were performed, and significant differences are indicated by different lowercase superscript letters (a, b, c).

**Table 3:** Distribution of transverse occlusal relationships according to vertical occlusal relationship in patients with class I malocclusion.

Transverse occlusal relationship	Open bite n (%)	Normal overbite n (%)	Deep bite n (%)	Total n	χ^2^	*P*	Cramer’s *V*
Non-crossbite	64 (43)a	337 (55.1)b	69 (65.7)b	470	261.303	<.001	0.223
Single-tooth posterior crossbite	43 (28.9)a	180 (29.4)a	29 (27.6)a	252			
Unilateral posterior crossbite	20 (13.4)a	55 (9)a	5 (4.8)a	80			
Bilateral posterior crossbite	22 (14.7)a	40 (6.5)b	2 (1.9)b	64			
Total	149 (100)	612 (100)	105 (100)	866			

Percentages represent the distribution of transverse occlusal relationships within each vertical occlusal subgroup of patients with class I malocclusion. Different lowercase superscript letters (a and b) indicate significant pairwise differences according to the Bonferroni correction (*P* < .05).

% = percentage, Cramer’s *V* = effect size, n = number of participants, χ^2^ = Pearson chi-square statistic.

**Table 4:** Distribution of transverse occlusal relationships according to vertical occlusal relationship in patients with class II malocclusion.

Transverse occlusal relationship	Open bite n (%)	Normal overbite n (%)	Deep bite n (%)	Total n	χ^2^	*P*	Cramer’s *V*
Non-crossbite	62 (38.5)a	399 (60.9)b	401 (85)c	862	157.935	<.001	0.231
Single-tooth posterior crossbite	44 (27.3)a	154 (23.5)a	46 (9.7)b	244			
Unilateral posterior crossbite	35 (21.7)a	79 (12.1)b	20 (4.2)c	134			
Bilateral posterior crossbite	20 (12.4)a	23 (3.5)b	5 (1.1)c	48			
Total	165 (100)	655 (100)	472 (100)	1288			

Percentages represent the distribution of transverse occlusal relationships within each vertical occlusal subgroup of patients with class II malocclusion. Different lowercase superscript letters (a, b, and c) indicate significant pairwise differences according to the Bonferroni correction (*P* < .05).

% = percentage, Cramer’s V = effect size, n = number of participants, χ^2^ = Pearson chi-square statistic.

**Table 5:** Distribution of transverse occlusal relationships according to vertical occlusal relationship in patients with class III malocclusion.

Transverse occlusal relationship	Open bite n (%)	Normal overbite n (%)	Deep bite n (%)	Total n	χ^2^	*P*
Non-crossbite	31 (20.4)	78 (28.5)	12 (27.3)	121	6.347	.385
Single-tooth posterior crossbite	25 (16.4)	48 (17.5)	4 (9.1)	77		
Unilateral posterior crossbite	25 (16.4)	52 (19)	12 (27.3)	89		
Bilateral posterior crossbite	71 (46.7)	96 (35)	16 (36.4)	183		
Total	152 (100)	274 (100)	44 (100)	470		

Percentages represent the distribution of transverse occlusal relationships within each vertical occlusal subgroup of patients with class III malocclusion. No statistically significant differences were observed; therefore, Bonferroni-adjusted pairwise comparisons were not performed.

% = percentage, n = number of participants, χ^2^ = Pearson chi-square statistic.

Within the class I malocclusion group (Table 3), the distribution of transverse occlusal relationships differed significantly according to vertical occlusal relationship (χ^2^ = 261.303, *P* < .001), with a small effect size (Cramer’s *V* = 0.223). Bonferroni-adjusted pairwise comparisons showed that significant differences were observed only for the non-crossbite and bilateral posterior crossbite categories. Specifically, the open bite subgroup showed a lower prevalence of non-crossbite and a higher prevalence of bilateral posterior crossbite than the normal overbite and deep bite subgroups, whereas no significant differences were observed for single-tooth or unilateral posterior crossbite.

Within the class II malocclusion group (Table 4), the distribution of transverse occlusal relationships differed significantly according to vertical occlusal relationship (χ^2^ = 157.935, *P* < .001), with a small effect size (Cramer’s *V* = 0.231). Bonferroni-adjusted pairwise comparisons showed that significant differences were observed across all vertical subgroups for non-crossbite, unilateral posterior crossbite, and bilateral posterior crossbite. Single-tooth posterior crossbite differed only between the deep-bite subgroup and the other 2 vertical subgroups.

Within the class III malocclusion group (Table 5), the distribution of transverse occlusal relationships did not differ significantly according to vertical occlusal relationship (χ^2^ = 6.347, *P* = .385). Bilateral posterior crossbite remained the predominant transverse occlusal relationship across all vertical subgroups, accounting for 46.7% of patients with open bite, 35.0% with normal overbite, and 36.4% with deep bite.

A multinomial logistic regression analysis was performed to evaluate the independent associations of age, transverse occlusal relationship, and vertical occlusal relationship with sagittal malocclusion (Table 6). The overall model was statistically significant (χ^2^ = 658.780, *P* < .001), explaining 25.5% of the variance in sagittal malocclusion classification (Nagelkerke *R*^2^ = 0.255). Age was not independently associated with sagittal malocclusion (*P* > .05). After adjustment for age, transverse and vertical occlusal relationships remained significantly associated with sagittal malocclusion. Compared with class I malocclusion, class II malocclusion was positively associated with deep bite (OR = 3.953, 95% CI: 3.103–5.036), whereas bilateral posterior crossbite (OR = 0.519, 95% CI: 0.348–0.775) and single-tooth posterior crossbite (OR = 0.652, 95% CI: 0.524–0.810) were less frequently observed. In contrast, class III malocclusion showed strong positive associations with bilateral posterior crossbite (OR = 10.218, 95% CI: 7.160–14.581), unilateral posterior crossbite (OR = 4.087, 95% CI: 2.829–5.905), and open bite (OR = 1.578, 95% CI: 1.175–2.118).

**Table 6:** Multinomial logistic regression analysis evaluating the independent associations of age, transverse occlusal relationship, and vertical occlusal relationship with sagittal malocclusion.

Comparison	Variable	OR	95% CI	*P*
Class II vs class I	Age	1.000	0.979–1.021	.996
	Bilateral posterior crossbite vs non-crossbite	0.519	0.348–0.775	.001
	Single-tooth posterior crossbite vs non-crossbite	0.652	0.524–0.810	<.001
	Unilateral posterior crossbite vs non-crossbite	1.153	0.847–1.568	.366
	Open bite vs normal overbite	1.056	0.820–1.358	.675
	Deep bite vs normal overbite	3.953	3.103–5.036	<.001
Class III vs class I	Age	1.019	0.995–1.043	.118
	Bilateral posterior crossbite vs non-crossbite	10.218	7.160–14.581	<.001
	Single-tooth posterior crossbite vs non-crossbite	1.175	0.846–1.632	.335
	Unilateral posterior crossbite vs non-crossbite	4.087	2.829–5.905	<.001
	Open bite vs normal overbite	1.578	1.175–2.118	.002
	Deep bite vs Normal overbite	1.233	0.826–1.840	.305

Model statistics: χ^2^(12) = 658.780, *P* < .001; Cox and Snell *R*^2^ = 0.222; Nagelkerke *R*^2^ = 0.255.

Odds ratios are adjusted for all variables included in the model. Reference categories were class I malocclusion, non-crossbite for transverse occlusal relationship, and normal overbite for vertical occlusal relationship. Odds ratios >1 indicate higher odds of belonging to the comparison group relative to the reference category.

CI = confidence interval, OR = odds ratio.

To evaluate the reliability and validation of the measurements, an additional analysis was performed using a representative subsample of 50 randomly selected patients. Agreement between AI-assisted and conventional manual overbite measurements was excellent (intraclass correlation coefficient = 0.925, 95% CI: 0.872–0.957). Inter-observer agreement was also excellent for both sagittal (κ = 0.906) and transverse (κ = 0.912) malocclusion classifications. These findings support the reliability of the AI-assisted overbite measurements and the reproducibility of sagittal and transverse malocclusion classifications.

## 4. Discussion

Epidemiological studies investigating the prevalence of orthodontic malocclusions are essential for identifying population characteristics, estimating treatment needs, enabling early intervention, guiding health policies, and predicting the population-level demand for orthodontic services.^[1,24]^ Numerous prevalence studies have been conducted worldwide, including in Türkiye, exploring differences by age, sex, and dentition stage.^[20–23,30,31]^ These investigations have typically assessed sagittal malocclusion, overjet/overbite, posterior crossbite, crowding or spacing, dental anomalies, and orthodontic treatment needs independently, rather than as components of an integrated occlusal system. However, despite the inherently multidimensional nature of malocclusion, few epidemiological studies have systematically investigated the interactions among sagittal, vertical, and transverse occlusal relationships.

Unlike previous prevalence studies that relied primarily on subjective clinical assessment of vertical discrepancies, the present study used an AI-assisted cephalometric tool to obtain standardized overbite measurements. Although the AI system served only as an assistive measurement tool, it provided objective and reproducible assessment across the entire dataset.^[26]^ To our knowledge, this is the 1st epidemiological study to integrate AI-assisted overbite measurements into a multidimensional analysis of malocclusion, thereby contributing a methodological advancement to malocclusion prevalence research.

The present study evaluated multidimensional malocclusion patterns across the entire treatment-seeking cohort; therefore, age and dentition stage were not analyzed as separate subgroups, consistent with the approach of Tsai et al.^[32]^ Although some studies have reported that the molar relationship in mixed dentition may change over time in relation to leeway space and sagittal jaw growth differences,^[33–35]^ others have suggested that early malocclusions do not tend to self-correct.^[36–38]^ Furthermore, a recent global meta-analysis reported comparable malocclusion prevalence across the primary, mixed, and permanent dentitions, supporting the rationale for the pooled analysis adopted in the present study.^[23]^

The present study showed that sagittal malocclusions were not equally distributed among treatment-seeking Turkish orthodontic patients (Table 1 and Fig. 2). Nearly half of the participants exhibited class II malocclusion (49.1%), followed by class I (33%) and class III (17.9%). No statistically significant sex differences were observed within sagittal groups, although the female-to-male ratio was slightly higher across all classes.

Previous epidemiological studies have reported wide variations in malocclusion prevalence across populations. Global systematic reviews found that class I ranged from 34.9% to 93.6%, class II from 4.4% to 44.7%, and class III from 1.4% to 19.4%.^[20,29]^ Meta-analyses further estimated worldwide prevalence rates at approximately 55.5% for class I, 24.7% for class II, and 10.7% for class III, with Europe showing the highest frequency of class II cases.^[1,23]^ In Türkiye, a recent meta-analysis reported prevalence rates of 56% for class I, 31% for class II, and 11% for class III.^[24]^ Local field studies in Türkiye revealed broader ranges, typically higher class I (39.4%–76.9%) and lower class II (11.4%–48.4%) and class III (3.5%–11.7%) than those observed in our cohort.^[18,25,39,40]^

However, studies limited to orthodontic populations rather than general samples reported rates more consistent with our findings – for example, Celikoğlu et al^[31]^ found 41.5%, 38.3%, and 16.7% for class I, II, and III, respectively, while Akbulut^[30]^ reported 44.6%, 44.8%, and 10.5%. Beyond Türkiye, orthodontic cohorts from Belgium, Nepal, Taiwan, and Korea also demonstrated higher class II (30.8%–63%) and class III (4.64%–36.1%) rates compared with school-based studies.^[32,41–43]^ Collectively, these comparisons indicate that treatment-seeking cohorts tend to concentrate severe malocclusions, whereas general population surveys usually report higher rates of class I. The higher prevalence of class II and III malocclusions in the present study is consistent with the treatment-seeking nature of the cohort, in which more severe malocclusions are overrepresented compared with community-based populations. Moreover, the type of malocclusion strongly influences treatment motivation, with severe class II cases particularly more likely to seek care than class III cases.^[44]^

The relatively high prevalence of class II and III malocclusions in our cohort can be attributed to the tertiary referral nature of our institution, which receives patients from Ankara and many surrounding regions, likely contributing to the higher proportion of complex malocclusions observed in the present cohort. This geographically diverse treatment-seeking population likely explains the elevated rates of severe malocclusions observed in our sample. Interestingly, when compared with a study conducted approximately 20 years ago at the same center, class II prevalence remained similar (49.3% vs 49.1%), whereas class I increased (27.5%–33%) and class III decreased (23%–17.9%).^[45]^ This difference may reflect changing esthetic expectations, improved access to orthodontic services, and increasing public awareness over time. Despite the inherent limitations of a treatment-seeking clinical cohort, the inclusion of patients from different geographic regions of Türkiye may enhance the representativeness of the findings for orthodontic referral populations.

Although sex differences were not statistically significant, females predominated numerically. This finding aligns with previous studies reporting no significant sex-related differences in malocclusion prevalence.^[18,21,24,25,30,31,46]^ Higher female participation has been attributed to both demographic factors and women’s greater attention to esthetics and oral health.^[41,47]^

When sagittal malocclusions were analyzed in relation to transverse traits, our findings demonstrated significant differences among class I, II, and III malocclusion groups. Unilateral and/or bilateral posterior crossbite was observed in more than half of class III patients (57.9% in total), a prevalence substantially higher than in class I (16.6% in total) and class II (14.1% in total). This pattern aligns with previous reports linking class III malocclusion to maxillary underdevelopment and transverse constriction.^[48–51]^ Previous morphometric studies have confirmed narrower intermolar and alveolar arch widths in class III patients compared with controls,^[9,37,52]^ suggesting that the high prevalence of posterior crossbite in this group may be related, at least in part, to underlying skeletal deficiency rather than solely to dental factors. However, the present study evaluated interocclusal relationships only and therefore cannot distinguish among dental, dentoalveolar, functional, and skeletal components. Clinically, these findings, together with previous evidence, suggest that class III patients may warrant careful transverse assessment, including evaluation of the potential need for maxillary expansion, particularly when posterior crossbite is present. Furthermore, the moderate effect size observed for the association between sagittal malocclusion and transverse occlusal relationship suggests that this association was not only statistically significant but also clinically meaningful.

In contrast, normal transverse relationships were most common in class II malocclusions (66.9%). Initially, this pattern may suggest a lower burden of transverse discrepancy in class II individuals. However, both classic and contemporary studies have emphasized the phenomenon of “masked transverse constriction,” in which maxillary narrowing is concealed by mandibular retrusion or dentoalveolar compensation.^[6,53]^ Recent studies of palatal morphology have confirmed that class II patients exhibit narrower maxillary palates and smaller palatal surface areas despite the absence of clinically evident crossbite.^[54]^ Similarly, morphometric studies have reported reduced intercanine, intermolar, and alveolar widths in class II division 1 individuals with no crossbite, compared with normal occlusions.^[53,55–58]^ Collectively, these findings may help explain why the prevalence of normal transverse occlusal relationships was high, whereas the prevalence of posterior crossbite was low in the class II malocclusion group. These observations indicate that clinical examination alone may underestimate transverse discrepancies in class II cases. From a treatment perspective, such “subclinical” constrictions highlight the importance of careful transverse assessment in class II patients, as underlying transverse discrepancies may otherwise be underestimated. Such findings may help identify patients who require further clinical evaluation.

In class I malocclusion, posterior crossbite was relatively uncommon and typically restricted to unilateral presentations (9.2%). The prevalence of single-tooth posterior crossbite was also significantly higher. Unilateral posterior crossbite and/or posterior premature contacts are often accompanied by functional mandibular lateral shift, as shown in multiple studies.^[59,60]^ In functional crossbites, mandibular deviation may result in asymmetric masticatory activity, midline shift, and temporomandibular joint adaptation.^[61]^ Such discrepancies should not be overlooked, as persistent functional shifts may predispose to skeletal asymmetry if left untreated.^[62]^ Therefore, even seemingly minor transverse discrepancies, particularly single-tooth posterior crossbite, should be carefully evaluated because they may indicate an underlying functional shift and may warrant further clinical evaluation.

Epidemiological studies examining the prevalence of transverse malocclusions are limited, and most have reported only the presence of posterior crossbite without considering sagittal or vertical relationships. In our study, the overall prevalence of posterior crossbite was 23%, which is higher than the 4% to 16% range reported in earlier Turkish prevalence studies^[19,25,31,63]^ and the ~7% to 9% estimated in global meta-analyses.^[1,20,23]^ Notably, Başçiftçi et al analyzed crossbite prevalence by sagittal classification, reporting 8.3% in class I, 12.1% in class II, and 52.9% in class III – which closely align with our current findings.^[18]^ These results emphasize that sagittal and vertical discrepancies, which are often overlooked, are key factors underlying the wide variation in posterior crossbite prevalence across studies.

Taken together, these results, supported by both effect size analysis and multivariable modeling, highlight that transverse occlusal relationships cannot be fully understood in isolation but should instead be interpreted within the context of sagittal and vertical malocclusion patterns. The high prevalence of posterior crossbite observed in class III malocclusion, together with previous evidence from morphometric studies, suggests that multidimensional assessment may provide a more comprehensive understanding of transverse discrepancies than evaluation of a single occlusal trait alone. Failure to recognize these multidimensional interactions may compromise long-term stability, underscoring the clinical value of integrated multidimensional assessment in both epidemiological research and clinical orthodontic practice.

When sagittal, transverse, and vertical relationships were analyzed simultaneously, significant differences were observed across malocclusion groups. In class I malocclusion, unilateral and bilateral posterior crossbite was relatively uncommon in patients with normal (15.5% in total) or deep bite (6.7% in total) but increased considerably in open bite cases (28% in total). In class II malocclusion, a clear gradient was identified: the prevalence of posterior crossbite rose as overbite decreased from deep bite (5.3%) to open bite (34.2%). Class III malocclusions demonstrated a different pattern: unilateral and bilateral posterior crossbite prevalence remained consistently high across all vertical subgroups, including deep bite cases (63.7% in total). These findings indicate that vertical discrepancies were strongly associated with transverse occlusal relationships in class I and class II malocclusions, whereas posterior crossbite in class III remained consistently prevalent regardless of the vertical occlusal relationship. Although statistically significant associations were identified within the class I and class II malocclusion groups, the corresponding effect sizes were small, suggesting that these transverse–vertical associations should be interpreted with appropriate clinical context. Collectively, these findings support the importance of evaluating malocclusion within a multidimensional framework rather than relying on a single occlusal plane.

These findings indicate that in class I and II malocclusions, reduced overbite is often accompanied by posterior crossbite, whereas deep bite tends to reflect preserved transverse harmony. Indeed, the prevalence of normal transverse relationships reached as high as 65.7% in class I and 85% in class II individuals with deep bite. These results underscore the importance of carefully evaluating transverse dimensions in patients with reduced overbite, where underlying transverse discrepancy may otherwise be underestimated. Although often overlooked in epidemiological studies, this highlights the importance of considering multidimensional interactions in the development of malocclusion.

From a broader biomechanical perspective, the observed vertical–transverse associations may be explained by previously proposed mechanisms involving masticatory musculature and craniofacial growth. Individuals with strong masticatory muscles – commonly linked with brachyfacial patterns and deep bite – have been reported to display greater transverse development, as increased muscular loading stimulates sutural growth and bone deposition.^[11,64–67]^ In this way, craniofacial growth may shift from a vertical to a more transverse pattern. Conversely, in hyperdivergent open bite patients, reduced elevator muscle activity has been associated with narrower maxillary morphology.^[68–72]^ Recent morphometric research further supports this, showing that class II patients with hyperdivergent vertical patterns have narrower palates and reduced surface areas, while hypodivergent class II individuals display broader palatal morphology.^[54]^ Such vertical–transverse interactions highlight the biomechanical underpinnings of the patterns observed in our study.

Overall, our results align with the existing literature in showing that deep bite is generally associated with preserved or enhanced transverse relationships, while open bite correlates with reduced transverse development. Importantly, the consistently high prevalence of crossbite in class III across all vertical groups, even with deep bite, underscores the complexity of multidimensional malocclusion patterns and the need for comprehensive multidimensional evaluation in diagnosis and treatment planning. This pattern may be consistent with previous reports describing maxillary skeletal underdevelopment in class III malocclusion; however, the present study did not directly assess skeletal transverse morphology. This interpretation is supported by previous research. For example, Chen et al found posterior crossbite to be inevitable in low-angle class III patients despite relatively greater arch widths.^[29]^ Clinically, these findings suggest that class III patients may warrant careful transverse assessment regardless of overbite status, including evaluation of the potential need for maxillary expansion when posterior crossbite is present.

To further explore whether these multidimensional associations were independent of potential age-related confounding, multinomial logistic regression analysis was performed (Table 6). After adjustment for chronological age, the observed associations remained significant, suggesting that the relationships among sagittal, transverse, and vertical occlusal characteristics were not explained solely by age differences. However, because dentition stage was not included in the regression model, residual developmental confounding cannot be completely excluded. Therefore, the regression findings should be interpreted within the context of the heterogeneous developmental stages represented in this treatment-seeking cohort. Deep bite showed the strongest independent association with class II malocclusion, whereas bilateral posterior crossbite demonstrated the strongest independent association with class III malocclusion. These findings are consistent with the value of a multidimensional approach to malocclusion assessment; however, the observed associations should be interpreted with consideration of the potential influence of developmental stage.

Taken together, these findings underscore that the vertical dimension is closely associated with transverse discrepancies and should not be overlooked during orthodontic assessment. These findings reinforce the value of integrated multiplanar evaluation for diagnosis, treatment planning, and epidemiological research.

Despite the large number of prevalence studies published to date, substantial heterogeneity persists in reported rates of malocclusion due to differences in geography, ethnicity, sample size, diagnostic criteria, and study design.^[1,23,29,32,42,43]^ Many of these investigations have focused on isolated occlusal traits – such as overjet, overbite, or posterior crossbite – rather than the multidimensional interactions among sagittal, transverse, and vertical relationships. Although orthodontic treatment adopts a holistic approach aimed at improving both function and esthetics of the dento-craniofacial complex, analyzing occlusal traits in isolation may overlook key multidimensional patterns. The present study addresses this gap by providing a multidimensional epidemiological perspective on malocclusion patterns.

The AI platform served exclusively as an assistive measurement tool, while all automated landmark identifications and measurements were verified and, when necessary, manually corrected by an experienced orthodontist before inclusion in the analyses. Integrating AI-assisted cephalometric measurement into epidemiological research represents a methodological advancement that enhances measurement standardization and reproducibility in large retrospective datasets. Furthermore, the excellent agreement between AI-assisted and conventional manual overbite measurements, together with the excellent inter-observer agreement for sagittal and transverse classifications, supports the robustness and reproducibility of the assessment protocol used in the present study.

Our findings demonstrate that transverse discrepancies should be interpreted together with sagittal and vertical occlusal characteristics rather than in isolation. In class I and II malocclusions, posterior crossbite became increasingly common as overbite decreased, underscoring the importance of evaluating the vertical dimension when diagnosing transverse discrepancies. Conversely, posterior crossbite remained highly prevalent across all vertical subgroups in class III malocclusion, which may be related to underlying skeletal transverse deficiency reported in previous studies. Clinically, these patterns may help identify patients who could benefit from further transverse assessment and individualized treatment planning, including evaluation of functional shifts in class I, possible transverse discrepancies in class II, and the potential need for maxillary expansion in class III patients.

Because the present cohort primarily comprised children, adolescents, and young adults, the present findings provide additional insight into multidimensional malocclusion patterns in treatment-seeking orthodontic populations. Although these observations may be relevant to the clinical assessment of growing patients, they should be interpreted as reflecting a treatment-seeking orthodontic cohort rather than the general population. Overall, these findings reinforce the value of comprehensive multidimensional assessment in both clinical orthodontics and epidemiological research. Our previous study reported the overall prevalence of sagittal, vertical, and transverse malocclusions and their association with sex using a larger cohort derived from the same clinical database.^[73]^ In contrast, the present study extends this work by focusing on multidimensional interactions among sagittal, vertical, and transverse malocclusion patterns. By incorporating additional analytical approaches, including effect size analysis, multinomial logistic regression, and reliability analyses, the current study provides insights beyond conventional prevalence reporting alone. Therefore, the present findings characterize multidimensional malocclusion patterns in a tertiary orthodontic referral population and may provide a foundation for future population-based epidemiological studies.

Future research should include longitudinal and multicenter studies incorporating developmental and skeletal variables to better understand the evolution of multidimensional malocclusion patterns and improve understanding of the timing of orthodontic intervention. Such studies may improve the generalizability of current findings beyond treatment-seeking orthodontic cohorts and further improve our understanding of multidimensional malocclusion patterns across different populations.

### 4.1. Limitations

This study has several limitations. First, because the study population consisted exclusively of treatment-seeking individuals attending a tertiary referral university hospital, the observed prevalence estimates are likely to overrepresent moderate and severe malocclusions and should not be interpreted as population-based prevalence for the Turkish population. However, Ankara’s status as the capital and a national referral hub – receiving patients from various regions and immigrant backgrounds – likely enhanced the heterogeneity of the cohort. Broader multicenter studies are warranted to confirm these findings on a population basis.

No subgrouping was performed according to age or dentition stage. Although chronological age was included as a covariate in the multinomial logistic regression analysis, adjustment for age may not fully account for developmental differences between mixed and permanent dentitions. Therefore, residual developmental confounding cannot be completely excluded. Future age- or dentition stage-stratified analyses may provide additional insights into developmental trajectories.

Transverse evaluation was limited to interocclusal relationships rather than direct arch width measurements, and skeletal cephalometric parameters were not included. While 3-dimensional imaging methods such as cone beam computed tomography would provide more comprehensive data, their routine use in large-scale epidemiological studies remains restricted by ethical and financial constraints. Future studies incorporating direct arch-width measurements and skeletal cephalometric variables may provide a more comprehensive understanding of the interactions between dental and skeletal components of malocclusion.

Although additional validation demonstrated excellent agreement between AI-assisted and conventional manual overbite measurements, AI-assisted assessment remains dependent on the quality of the original radiographs and may still require clinician verification in challenging cases.

Although selection bias is inherent in a treatment-seeking orthodontic cohort, the consecutive inclusion of all eligible patients with complete standardized records and the application of predefined eligibility criteria minimized the potential influence of selection bias and missing data on the study findings.

## 5. Conclusion

This large-scale study evaluated sagittal, vertical, and transverse malocclusion traits in a treatment-seeking Turkish orthodontic population. By analyzing these 3 dimensions together, it revealed clinically meaningful interactions that are often overlooked in conventional prevalence studies.

- Class II malocclusion (49.1%) was the most prevalent sagittal pattern, followed by class I (33.0%) and class III (17.9%).
- Normal transverse relationships predominated in class I and II; however, posterior crossbite was strongly associated with class III, affecting more than half of the patients regardless of vertical pattern.
- In class I and II, posterior crossbite was significantly more frequent in open bite cases and least common in deep bite, underscoring the association between vertical and transverse occlusal relationships. In contrast, class III malocclusions exhibited persistently high posterior crossbite prevalence across all vertical subgroups, regardless of overbite status.

These findings demonstrate that malocclusions are multidimensional conditions rather than isolated deviations, as sagittal, vertical, and transverse discrepancies frequently coexist and show complex interrelationships. The integration of AI-assisted vertical assessment, combined with a large sample size and structured multidimensional assessment, provides a more refined understanding of occlusal complexity and enhances the reproducibility of epidemiological research.

From a clinical standpoint, the findings highlight the importance of comprehensive multidimensional assessment and individualized treatment planning. Beyond clinical orthodontics, this study provides valuable evidence regarding multidimensional malocclusion patterns in a large treatment-seeking orthodontic cohort and may generate hypotheses for future population-based epidemiological studies.

## Acknowledgments

The statistical analysis was performed under supervision of the institutional biostatistics unit. The authors used OpenAI’s ChatGPT to assist with language editing and manuscript refinement. All scientific content, analyses, interpretations, and final editorial decisions were performed and verified by the authors.

## Author contributions

**Conceptualization:** Merve Berika Kadıoğlu, Nazlı Türkel, Meliha Rübendiz.

**Data curation:** Nazlı Türkel.

**Formal analysis:** Merve Berika Kadıoğlu, Nazlı Türkel.

**Funding acquisition:** Merve Berika Kadıoğlu.

**Investigation:** Merve Berika Kadıoğlu, Nazlı Türkel.

**Methodology:** Merve Berika Kadıoğlu, Nazlı Türkel, Meliha Rübendiz.

**Project administration:** Merve Berika Kadıoğlu, Nazlı Türkel, Meliha Rübendiz.

**Resources:** Merve Berika Kadıoğlu, Nazlı Türkel.

**Software:** Nazlı Türkel.

**Supervision:** Meliha Rübendiz.

**Validation:** Merve Berika Kadıoğlu, Nazlı Türkel.

**Visualization:** Merve Berika Kadıoğlu.

**Writing – original draft:** Merve Berika Kadıoğlu, Nazlı Türkel, Meliha Rübendiz.

**Writing – review & editing:** Merve Berika Kadıoğlu, Nazlı Türkel, Meliha Rübendiz.
